# The Use of a Detection Dog to Discriminate Between Moss Species to Increase Coverage for Reporting the Presence of a Cryptic Species

**DOI:** 10.3390/ani16213539

**Published:** 2026-11-09

**Authors:** Patrice Kerrigan, Niamh Carmichael, Tanja Karpela, Kate Moodey, Richard Weyl, Caroline Finlay

**Affiliations:** 1 Conservation Detection Dogs NI, Newtownards BT23 5NN, UK; patrice@cddni.com (P.K.); kmoodey01@qub.ac.uk (K.M.); 2 Centre for Environmental Data and Recording, Belfast BT9 5AB, UK; niamh.carmichael@nationalmuseumsni.org; 3 International K9 Institute, 25500 Salo, Finland; info@ik9i.com; 4 School of Biological Sciences, Queen’s University Belfast, Belfast BT9 5DL, UK; 5 Northern Ireland Environment Agency, Belfast BT7 2JA, UK; richard.weyl@daera-ni.gov.uk

**Keywords:** *Hamatocaulis vernicosus*, detection dog, ecology dog, bryophyte, moss, Habitats Regulations Reporting, slender green feather-moss

## Abstract

There has been a rise in the use of conservation detection dogs (CDDs); however, they are yet to be noted in science for bryophyte detection. If implemented effectively, this method could have a significant positive impact, particularly due to the need for specialists in the identification of these species. This study aims to determine whether a CDD can be imprinted on a moss species, specifically *Hamatocaulis vernicosus,* the slender green feather-moss, and used to increase survey coverage in Northern Ireland. To test the capability of the dog on this new odour, a lab-based discrimination trial involving double-blind line-ups was conducted, including moss species that occur in similar habitats and are closely related to *H. vernicosus*. After the discrimination trial, the dog completed field trials in an area where the species is known to be present. Results show that the dog had 100% accuracy when completing the discrimination trial, showing the dog’s ability to discriminate between the target moss and other moss species used. During the field trial, an indication and positive confirmation of species were achieved within 1 min 34 s of searching. CDDs are clearly on the way to becoming a useful tool in bryophyte projects or projects focussing on other Habitats Regulations Reporting-annexed species, particularly those that are cryptic and time-consuming to survey.

## Simple Summary

Conservation detection dogs (CDDs) are highly trained scent detection dogs that have been used in science, government work and law enforcement for many years and with many odours, but they have yet to be utilized for moss detection, as recorded in science. The aim of this project was to see if one such dog could be used to detect and find a specific moss species, the slender green feather-moss. First a lab trial tested the discrimination abilities of the dog. During this trial it had to identify the correct moss out of other similar mosses in a line-up situation with no influence from the handler or tester. After 100% accuracy was obtained, a field trial was then undertaken where the dog was taken to a site with known slender green feather-moss presence, but with the specific areas of moss presence unknown. In this trial the dog successfully found the moss (which was confirmed by an expert) after 1 min 34 s of searching. This project shows how conservation detection dogs could successfully be used as time-reducing, accuracy-increasing methods in the field of bryophyte identification or for other similar cryptic species.

## 1. Introduction

Dogs are well established in detecting bombs and narcotics, as well as in search and rescue services, due to their olfactory abilities. A dog’s nose has more than 220 million olfactory receptors compared to humans, with 5 million, and their brains are dominated by their olfactory cortex [1] (pp. 183–222). In addition, they have the added benefit of having an organ, especially for olfactory use, called the Jacobson’s organ. This, combined with the nose remaining moist and cool, results in a tool well suited to detecting minute changes in odour [2] (pp. 1–14).

This affinity for scent detection has also been harnessed for conservation since 1890 in New Zealand, starting with kiwi (*Apteryx* spp.) searches [3] (p. 96). We are now seeing a rise in projects involving conservation detection dogs in the UK with successful results, such as great crested newt projects, [4] (pp. 6–15) bat and bird carcass detection around wind turbines, [5] (pp. 852–864) and many others [6] (pp. 568–579). These dogs are highly trained and can detect scent at distance and depth, and they have been proven to be more effective at searching than humans in most studies [6] (pp. 568–579), [7,8] (pp. 34–40).

In a recent study, it was found that, compared to animals, there have only been a small number of plant species detected using dogs, the numbers being 408 animals, 42 plants, 26 fungi, and six bacteria species [6] (pp. 568–579). Of these 42 plant species, bryophytes (hornworts, mosses and liverworts) were not listed. Aquatic and semi-aquatic studies on other species have been conducted, ruling out the environment as a reason for this lack of research [9] (pp. 1–23); however, the complex life stages and the physical makeup of bryophytes could be a factor, with the organisms lacking roots and requiring water for sexual reproduction as well as asexual physical fragmentation [10].

In Northern Ireland it is a requirement to monitor and report on selected species’ conservation status every six years under Habitats Regulations Reporting (HRR) (previously known as Article 17 of the Habitat Directive). One HRR-annexed species requiring reporting is *Hamatocaulis vernicosus*, with the common names of slender green feather-moss or varnished hook-moss. *H. vernicosus* is a vulnerable cryptic bryophyte species with particular habitat requirements, making it more susceptible to environmental threats. This is especially concerning for *H. vernicosus*, given that neutral flushes (areas where underground water flows onto the surface of wetland areas) running through fen (peat-accumulating wetland areas) meet its specific requirements and are habitats in a precarious state in the UK and Ireland [11]. The presence of *H.vernicosus* on managed wetland sites with the addition of spring water is a positive indicator of low pollution levels and good management from landowners [12]. *H.vernicosus* is widespread throughout Europe but in scattered numbers, and it can often be mistakenly identified as *Scorpidium cossonii* [13] and *Warnstorfia exannulata* [11]. In the latest IUCN red list report, *H.vernicosus* has been assigned vulnerable status, and it is showing a depleting population trend [14], making effective monitoring methods even more critical, especially in Northern Ireland and the Republic of Ireland with their already dwindling and sparse populations.

*H. vernicosus* was reported in four 10 km grid squares in Northern Ireland within the last reporting period [15]. Using the Irish grid reference system these include the 10 km squares D30 (reported in 2011), J24 (reported in 2012), J34 (reported in 2012) and H88 (reported in 2012). The species was recently rediscovered at one new locality, in square H13, due to increased surveillance and identification effort rather than an expansion of the moss’s range. This moss is particularly cryptic as it looks similar to a number of other hook-mosses in the same environment. There is also a lack of expertise in identification of this moss, making it difficult to increase human survey effort.

### Aims and Objectives

The aim of this study is to determine whether a conservation detection dog can be trained to identify a specific moss species, namely *H. vernicosus*, to help increase survey coverage of this species in Northern Ireland. The objective is to train the dog to recognise and distinguish the target moss species from other moss species from the same environment. The dog’s ability to identify moss in its natural habitat will then be tested by conducting searches in an area in which *H. vernicosus* is known to be present and verifying the dog’s finds with a bryologist. Additionally, this study aims to investigate the success rate of dogs in other worldwide moss detection projects, if any exist.

## 2. Materials and Methods

### 2.1. Imprinting and Proofing

Target moss samples (*H. vernicosus*) and mosses from the area surrounding the target species were collected using nitrile gloves and glass jars. The dog was trained using the Kong^®^ method, in which dogs are trained to detect various sizes of Kong^®^ (a red rubber dog toy); the Kong^®^ was then placed with the new target odour in a pot with a number of other pots in a line and gradually reduced in size until the Kong^®^ was fully removed and the dog is indicating solely on the target odour itself. No more than 3 training sessions a day, 3 times a week were performed, more due to handler time constraints than the ability or welfare of the dog. A session never exceeded 20 min, as this is what has been found to work best with this particular dog. This method is known to enable quick and accurate imprinting and has been used extensively in mine detection dog training as referenced by Norwegians Peoples Aid [16], conservation detection projects [17], and the National Police of the Netherlands [18].The way the dog indicates the presence of the target odour to the handler is by performing a behaviour that the handler has chosen to represent an indication, usually sit and stare, stand and freeze, or lie down.

Once the dog consistently indicates on the target moss without Kong^®^ being present, i.e., three consecutive correct indications, the proofing process begins. Proofing involves refining the odour picture for the dog, verifying that the dog is indicating solely on the target odour. In this stage, the other pots are filled with distractor odours. To begin with, these distractors consist of possible contamination odours, i.e., gloves, empty glass jars, Ziploc bags. Once the dog can confidently identify the target odour within this line-up (three consecutive correct indications), a moss species from a completely different habitat, unrelated to the target moss, is included in the line-up. Once the dog confidently identified the target moss within this line-up, the next step was to include samples from the environment where the target moss was collected: a water sample, grass, reeds/rushes, and, finally, other moss species.

Once the dog consistently identified the target species within a line-up containing other moss species and distractors, the target moss was moved outside. A known sample of the target was placed out in an area without any competing smells from its original environment; when this was successful a sample that had not been used for previous training was put out. Moving to the environment where the moss has been recorded, this was repeated, and then finally a search for the actual moss in situ was conducted to prove that the dog is able to find the moss in the environment, with any finds verified by a bryologist.

### 2.2. Discrimination Trials

The discrimination trials consisted of a line-up of 6 pots. These trials were designed to show that the dog could consistently identify the target sample among a collection of other mosses in a double-blind set up. The mosses selected occur in the same area as the target species. They include *Warnstorfia exannulata*, *Scorpidium revolvens*, *Rhytidiadelphus squarrosus*, *Calliergonella cuspidata* and *Sphagnum palustre*. A random selection of these mosses and possible contamination odours was used during the trials. If the dog correctly passed over a species at least three times and selected the target species each time, the discriminator was removed and replaced with a new discriminator species.

A technician arranged the pots and the handler walked the dog around the pots until the dog paused and held its indication position for a minimum of five seconds. If the dog paused for less than five seconds and then continued, this is not considered an indication. The handler did not know the location of the target species and continued walking to the end of the line even when the dog paused and indicated. The technician was also out of the dog and handler’s eyeline, behind an object, so as not to influence the dog in any way. The dog is only allowed two passes of the pots during the trial. If the dog did not indicate the target moss on the second pass, it was considered a miss. Three blank trials were included in which the target moss was not present to prove that the dog was not indicating on a pot at random. This method was selected based on Porrit et al. 2015 [19] and was later used by the National Protective Security Authority [20].

### 2.3. Field Trials

Field trials took place in an area in which *H. vernicosus* was known to be in situ, namely, in Slieve Croob, Co. Down. The dog was started at two spots downwind in the area and the time taken for the dog to give an indication was recorded. The coordinate of the indication was taken, and samples and photos from the area were also taken. The location coordinates and photos were then passed on to a bryologist to verify the find when they returned to the area.

### 2.4. Other Moss Detection Projects

A callout on social media was sent out on 07/08/2023, and emails were sent to the larger detection dog organisations to find other projects that have used dogs in this way. An interview was conducted with any respondents and included questions on sample collection, dog selection, imprinting, search style, and discrimination (the full list of questions can be seen in Appendix A).

## 3. Results

A checklist of variables from Mckeague et al. 2024 [7] was completed. This can be seen in Appendix B. Appendix B gives further details on dog selection, training and testing samples, team experience and previous odours.

### 3.1. Dog Selection

**Figure 1:**
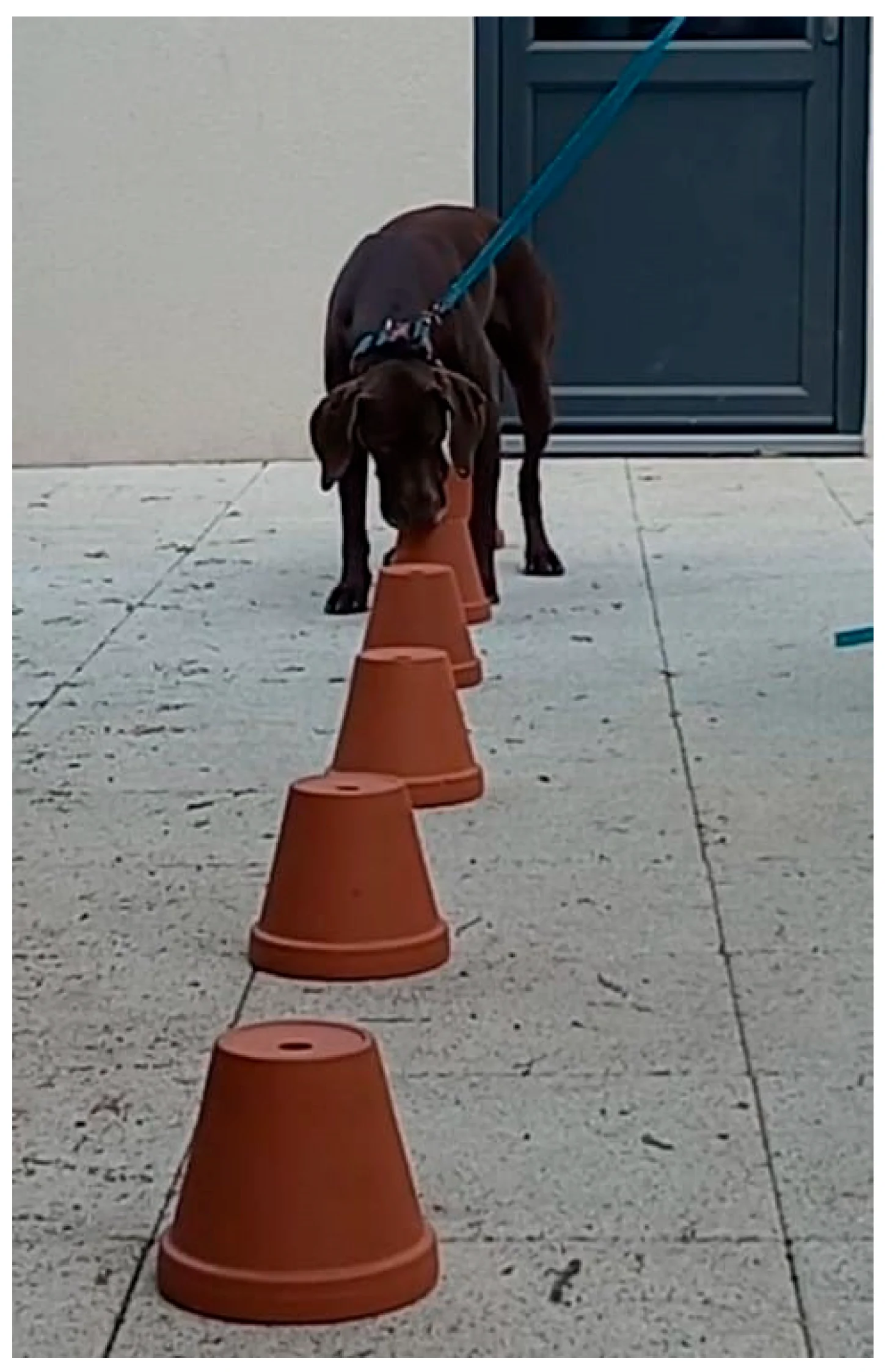
Photograph showing the line-up consisting of 6 pots. The dog was walked along the line of pots containing the target odour or a distractor/discriminator odour. This trial is double blind with handler and dog not knowing where the target odour is and the tester hidden from view.

The dog picked for this work was “Ziba”, a five-year-old German Shorthaired Pointer (Figure 1) that had been working in conservation detection for over a year, with her handler and trainer Dr Caroline Finlay, and in explosive detection before that in the Netherlands. Ziba’s indication that she has found odour is to freeze and point towards the source of the odour.

### 3.2. Imprinting and Proofing

The collection of the target moss, other plants and distractors from the environment was conducted on 25 January 2023. The samples were collected by the dog handler in the presence of a bryologist using gloves and jars that had been sterilised by boiling. Samples in jars of the same species were held in a Ziploc bag together to remove possible contamination from other samples. These were then stored in an insulated, unheated building that keeps a consistently cool temperature. Imprinting of the moss began on the 29th of January. By the 1st of February the dog was indicting on the moss without Kong^®^ present and indicating on samples that it had not met before in training.

Proofing of the odour began on the 6th of February with gloves, empty jars and Ziploc bags to begin with. The dog successfully passed over these distractor odours and identified the target moss. On the 13th of February, the dog was tested on other plants and water from the environment, including grasses, reeds/rushes and flush water samples. The dog successfully passed over these distractor odours on the second try and identified the target. To ensure the dog was not confusing the target moss with other vegetation, the dog was taken to the field to be used for the field trials and directed to search. It was possible that the dog may have indicated on another hook moss, *W. exannulata,* so this species was added to the proofing list.

In total, five other moss species were used for proofing; these included *W. exannulata*, *S. revolvens*, *R. squarrosus*, *C. cuspidata* and *S. palustre*. The only moss that the dog struggled with at the proofing stage was *W. exannulata*. The dog indicated on this a number of times during the proofing stage. To combat this and possibly correct contamination of training samples, all samples of *H. vernicosus* were replaced with new samples that had been checked to ensure that they did not contain any pieces of *W. exannulata*, and new samples of *W. exannulata* were collected to ensure that they were also free from contamination by the target moss. By 27 February 2023, the dog was able to discriminate between all moss samples and select the target species. At this point discrimination trials were able to proceed.

### 3.3. Discrimination Trials

In total, 15 trials were conducted using a six-pot line-up; these trials were conducted over 2 days (Figure 1).

After the dog correctly identified the target moss species a minimum of three times when it was present in a line-up with a discriminator moss, the discriminator moss was replaced with another species. The species used in each trial and the distractors used are listed in Table 1.

Out of the 12 trials with the target moss included, the dog correctly indicated on the target species 100% of the time and never indicated on a pot containing a distractor odour. This means that the dog did not indicate on any other moss species from that habitat or a possible contamination odour. During the three blank trials, the dog correctly did not indicate on any of the pots.

### 3.4. Field Trials

A suitable habitat area was mapped within the area known to have *H. vernicosus*. Two starting points on the edge of this area were identified to begin the searches. The dog was worked on a longline due to livestock. The time for each search was recorded until the dog indicated. The place at which the dog indicated was recorded on a GPS device, and photographs and samples of possible target mosses were collected.

**Figure 2:**
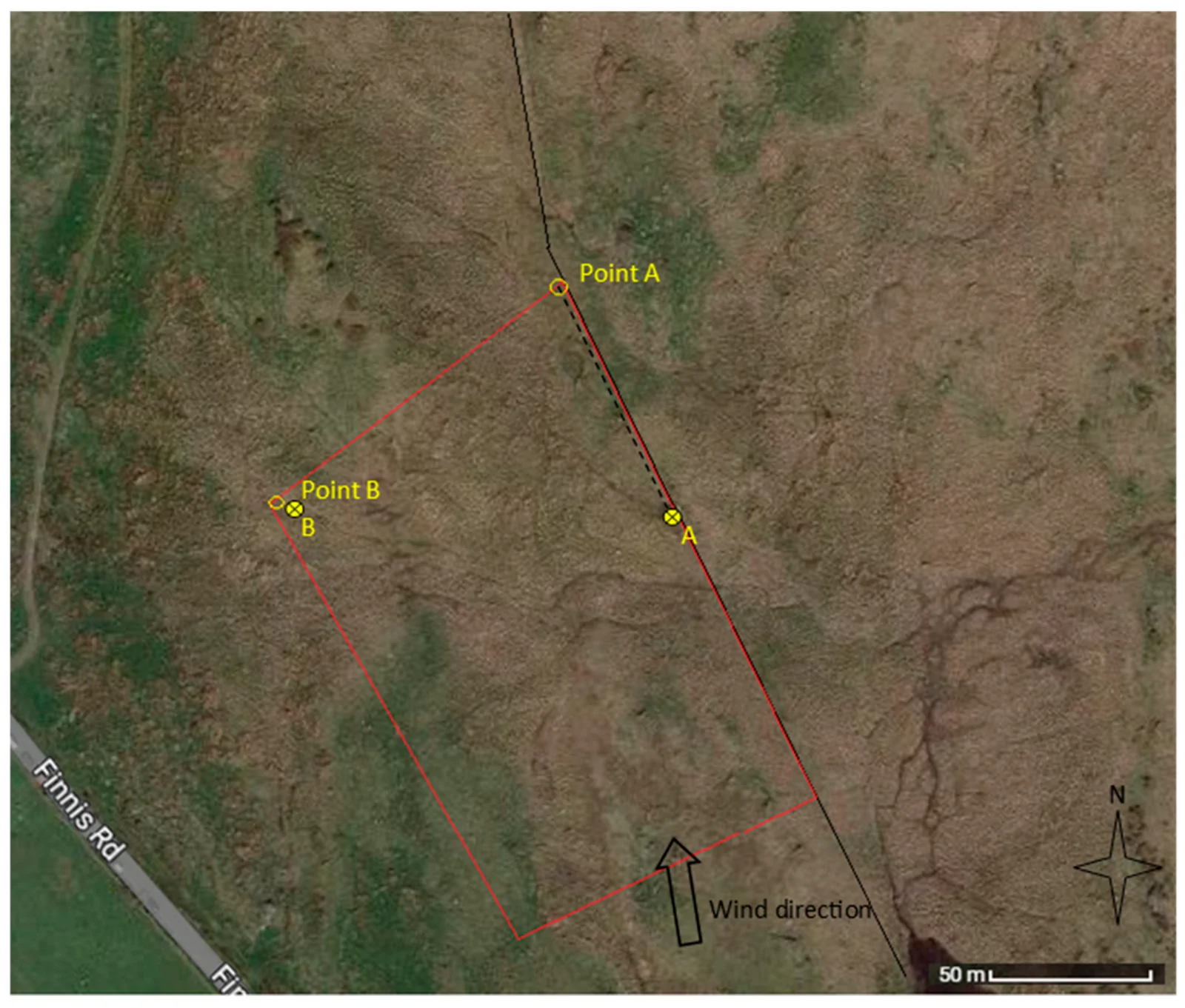
Shows a map identifying the starting points for the dog’s searches (yellow circles), the search area (in red outline) and the fence line (in black). A black arrow identifies the wind direction on the search day. The points at which the dog showed an indication or interest are represented by closed circles. Aerial imagery from Google Earth 2025; data provider Airbus.

**Figure 3:**
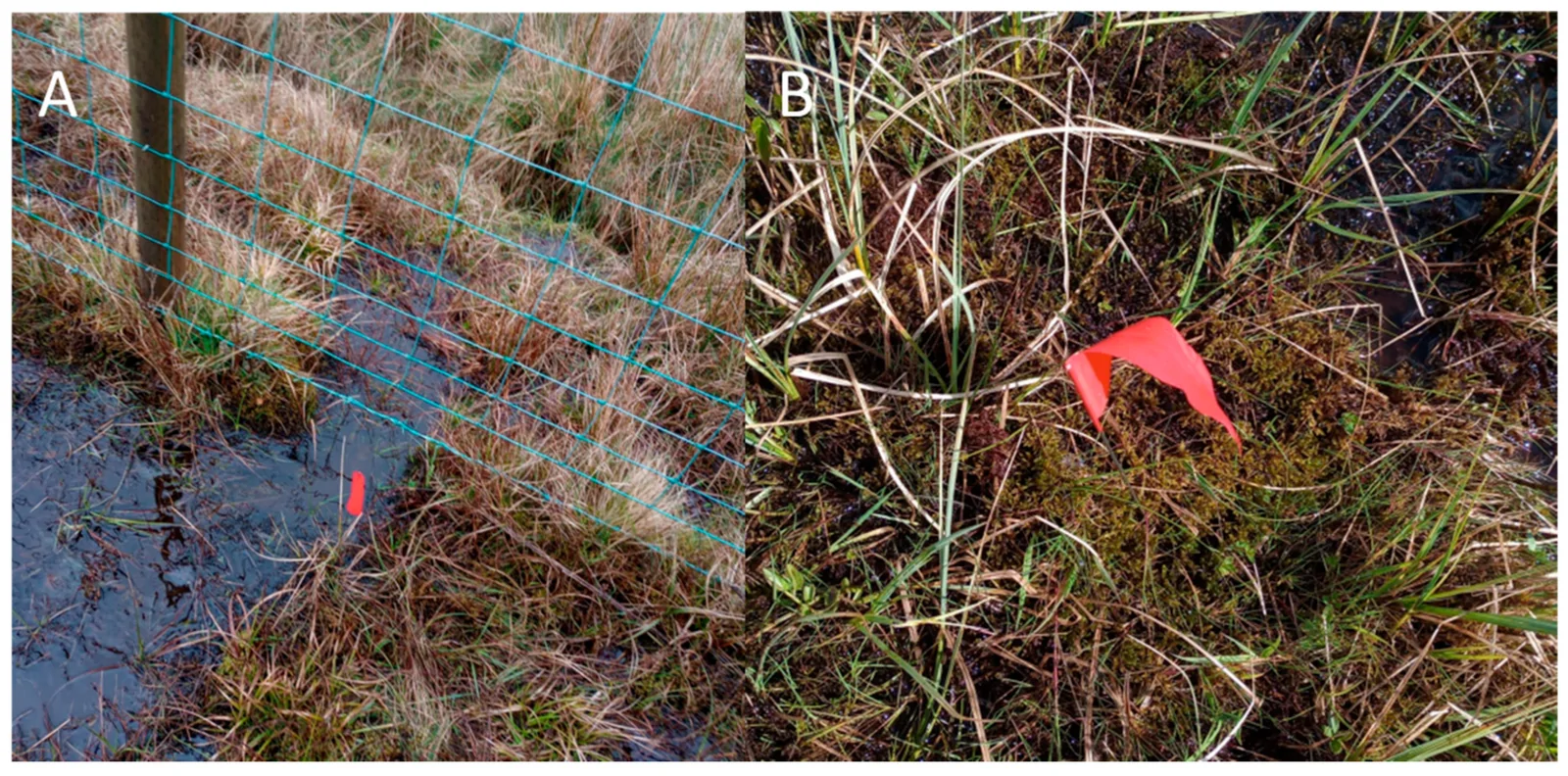
Shows the two areas indicated by the dog: (**A**) is the area found from start point A, and (**B**) is the area identified from start point B.

From start point A (Figure 2), the dog indicated after 22 min; from start point B, the dog indicated within 1 min 34 s. Photographs of the areas indicated by the dog are presented in Figure 3. Verification of these finds occurred on the 26th of May and was performed by Richard Weyl, NIEA. At indication A, no *H. vernicosus* was identified, but the area was within 5 m of the target species and along a fence line; indication B was in the open and *H. vernicosus* was identified exactly where the dog indicated. No habitat was deemed suitable for *H. vernicosus* before indication point A or the area between point A and B, so it was unlikely the dog had missed any areas of the moss before this indication.

### 3.5. Other Moss Detection Projects

There was only one respondent successfully recruited for this part of this study; this is believed to be representative of the number of moss detection projects worldwide currently. This one unpublished project was completed in Salo, Finland, through the International K9 Institute, which used detection dogs to find green shield-moss. In 2020, the *Buxbaumia viridis* (Br.) (green shield-moss) project focused on an endangered and rare moss species in Finland (T. Karpela, personal communication, 21 August 2023). This moss is similar to *H. Vernicosus* as it is difficult to identify and there is a lack of data recorded on this species. A specialist conservation dog trainer and handler, Tanja Karpela, worked with a German shepherd and a bryologist on the project. The dog was first trained on the odour of the sporangium, which is defined as the part of the moss that produces spores [21]. However, green shield-moss sporangium is so small it was too difficult to find. Karpela (personal communication, 21 August 2023) then tried to collect the protonema (defined as the spore growth that will later develop into leaves [21] of the moss) to see if the dog could be trained that way. Unfortunately, when collecting it in the field, there was cross-contamination between different mosses meaning that the dog could not associate one odour to the target moss (T. Karpela, personal communication, 21 August 2023). The rhizoids (defined by the later development of the protonema [21]) of the moss were grown on agar medium under laboratory conditions by a university researcher and this was the scent used to train the dog to detect the target moss (T. Karpela, personal communication, 21 August 2023). Time was then spent on scent discrimination trials (line-ups) to train the dog to indicate on the rhizoid and not the agar itself. Finally, the dog was taken into the field to detect green shield-moss. The project was deemed successful because the dog was able to detect this rare moss and ignore other mosses and environmental factors in the field (T. Karpela, personal communication, 21 August 2023).

## 4. Discussion

The results of the line-up discrimination tests show that detection dogs can discriminate between moss species; however, this is dependent on the quality of training samples. This proved to be the case in this study, when the dog was imprinted again on the correct sample that had been checked by a bryologist for contamination and did not struggle to correctly indicate on the target moss, and in the study completed in Finland in which it was found that samples grown in the lab, which therefore had no opportunity to be contaminated, worked best for imprinting. This is a common phenomenon within the detection dog industry and guidance notes on how to control contamination have been produced to deal with these issues [22].

During the discrimination trials the dog scored 100% accuracy with no false alarms. There was no hesitation with these indications, which were held for over 5 s, showing that the dog could easily discriminate between moss samples. There is some confidence in this as all samples, target or non-target, were collected on the same day, stored in the exact same way and clean pots were rotated in during the discrimination trials so the target species were not always in the same pot. However, this was only done with one dog in this study so it needs to be tested with further dogs to see if this is an easy odour for all dogs, or is Ziba particularly good at discrimination. The fact that there has been another dog trained successfully in Finland does give us some confidence this is not a one-off.

The use of the dog as a reporting tool is also demonstrated in this case study. The dog correctly identified an area of moss within minutes. This could then be verified by an expert on site quickly. However, it is important to note that this was a small search area and in a known area of the target species. A more realistic search in an applied setting with an unknown population of the target species would give a better appreciation of how useful the dog would be in this context. The presence or absence of the species is what is recorded during reporting in each 10 km square. Although there were only two field trials, this would be representative of how known presence could be reconfirmed for reporting. For unknown presence, for example in a square that has never been searched before, a different search strategy may be necessary. This could take the form of transects through habitat deemed suitable, but with closer transects than those chosen for the field trials here.

It is important that either a species expert attends the site with the dog team or that the handler is upskilled to accurately identify indications, the former being preferable. In the field trials, one area where the dog indicated (indication A) did not have the target moss present. The interest shown here by the dog could be for a number of reasons: the dog is cueing off of the habitat, the handler “talked” the dog into the indication or the moss was within a certain distance of the indication site and the odour was pooling on the fence line. This particular dog has been trained to indicate at the first presence of the odour and then slowly move in to pinpoint, as this is particularly useful for curlew nest identification which the dog had also been in training for at this time; however, in this case it could be detrimental as the handler did not give the dog enough time to pinpoint and took the first sign of interest as the point of indication. It is believed that this is what happened in this case, as the dog did not incorrectly indicate on any other areas within that habitat, and the handler has performed several drills to demonstrate that the dog did not cue off of the handler’s movements in training. The wind direction during that specific day would also be conducive to this happening. In future, a dog that is able to pinpoint directly would be more suitable for this project if the goal is to identify areas of target moss quickly and accurately. If there had been a bryologist on site or the handler had been upskilled in moss identification for this search, it would have been known that the dog was “incorrectly” indicating and either needed to move on from the area or work into the odour fully to pinpoint. Eventually, a dog and handler become experienced enough in searching for the specific species that a bryologist would not be needed for every search and would only be required to ensure the competency of the dog and handler team for each season or project.

Calculating the accuracy of the dog, to show how successful the dog is in finding the target moss in an area, would be the next step for this study and comparing it to human-only teams, to prove that training and hiring a dog for this work is cost-effective. If the dog can pinpoint areas and a person can attend afterwards to verify, or the handler is upskilled in accurate species identification, then using a dog for detection would ensure time-efficiency for survey teams, particularly at a national scale. In many studies dogs have been found to be faster and more accurate at detecting a variety of species than human searchers [8]. This has not been determined for a moss species yet, but the results of this study showing that the dog does not struggle to discriminate between moss species and can locate it in a suitable area are very promising. It would also be important to consider the variation between dogs, depending on their previous training history and experience, as Ziba was selected because of her previous successes in the field.

One of the issues that occurred regularly during the field trial period was heavy rain and snow covering the moss species. It has not been shown if a dog can identify a moss species when it is covered by water or a layer of snow, and it was decided not to add this to the dog’s repertoire during this short study period. However, there are several studies in which dogs have searched for odours underwater, especially in the search and rescue field, as well as in the field of conservation, e.g., searching for invasive species such as mussels [9]. In Deshon et al. (2016) [23], dogs were very quickly (within 3 days) taught to indicate on Quagga mussels at varying concentrations in water. This additional capability would enable a dog team to identify areas throughout the winter when human-only teams are unable to identify moss species visually and is an area for future research.

## 5. Conclusions

A dog has the potential to be a great asset to a team seeking to determine the range of a moss species, especially when the species is hard to identify visually. Further research is required as to the competency in larger areas, including those in which the moss is not already known to be present. The species that would benefit most from the conservation detection dog method are those that are difficult to find visually, i.e., hidden under water or vegetation, well camouflaged or incredibly small, but able to spread over a large area, and those that are difficult to distinguish from other species [1]. These species are often under-recorded with insufficient data to determine their short-term or long-term population trends. Other possible HRR-annexed species that could benefit from a detection dog as a survey method are: the Geyer’s whorl snail (*Vertigo geyeri*), the narrow-mouthed whorl snail (*Vertigo angustior*), the Desmoulin’s whorl snail (*Vertigo moulinsiana*), the freshwater pearl mussel (*Margaritifera margaritifera*), white-clawed crayfish (*Austropotamobius pallipes*), and Killarney fern (*Trichomanes speciosum*). All of these species are time consuming to survey and are cryptic in nature.

## Figures and Tables

**Table 1:** Illustrating the discrimination trials conducted on various species of non-target moss, possible contamination odours and the target moss with the resulting indications. All pot positions were randomised. (HV = *Hamatacaulis vernicosus*, SP = *Spaghnum pulstre*, RS = *Rhytidiadelphus squarrosus,* SR = *Scorpidium revolvens,* CC = *Calliergonella cuspidate,* WE = *Warnstorfia exannulata.*).

Trial	Samples Within Trial	Correct Indication?
1	Gloves, Ziplocs, SP, HV	Yes
2	Gloves, Ziplocs, SP, HV	Yes
3	Gloves, Ziplocs, SP, HV	Yes
4	Gloves, Ziplocs, SP	Blank
5	RS, SP, Ziplocs, HV	Yes
6	RS, CC, Ziplocs, HV	Yes
7	RS, CC, Gloves	Blank
8	RS, CC, Gloves, HV	Yes
9	WE, CC, Gloves, HV	Yes
10	WE, Gloves, HV	Yes
11	WE, Gloves, SR	Blank
12	WE, Gloves, SR, HV	Yes
13	SR, Gloves, HV	Yes
14	SR, Gloves, HV	Yes
15	SR, Gloves, HV	Yes

## Data Availability

The original contributions presented in this study are included in the article. Further inquiries can be directed to the corresponding author.

## Appendix A

Questions to other dog teams

Verbal consent statement: Thank you for agreeing to take part in this interview. The purpose of this interview is to gather information, experiences and opinions relevant to the project.

Your participation is entirely voluntary. You are free to decline to answer any question or withdraw from the interview at any time without providing a reason.

Information collected during the interview will be used solely for the purposes of this project. Any information shared will be treated confidentially, and personal details will not be disclosed without your explicit permission. You will be given the opportunity to review all publications created from the data created from this interview.

1. Introduction to project

What was the project called:

Who were the project partners and what was your job on the project:

What was the aim of the project:

What was the aim of using a dog on the project if different from the overall project aim:

2. Dog selection

How did you select the dog or dog team you were working with:

Was there anything you would have preferred that dog or dog team to have or be able to do:

3. Imprinting odour

What species was the target odour:

How did you imprint the dog and on what:

Who gathered the samples used to imprint the dog:

How easy was it to get samples to imprint on and how did you store them for longevity:

Would you have done anything differently if you do the project again:

Did you perform discrimination trials, if so, what did you use as distractor scents and what was the outcome of these trials:

4. Search style and operational searches

What search style and indication did the dog have:

Was there any particular training you gave the dog for this project:

Is there any particular training you would focus on if running the project again:

Did you perform field trials before making the dog operational, if so, how was this done:

When the dog was on an operational search how did you check if the dog indicated correctly, did you reward right away or did you check then send the dog back in to reward:

5. Search site

Where was the search site and can you describe the area:

How long would you be on site for:

Were there any welfare issues for the dog on the site or health and safety issues you had to take into account working on that site:

6. Communication between scientist and dog team

How was the communication between dog team and ecologist:

Was there anything you wish you had known before the searches began or being on site:

Would there be anything you will make sure to communicate promptly if a project goes forward again:

7. Welfare concerns external to the team

Were there any welfare concerns for the target species that you took into account:

Were there any welfare concerns for non-target species that you took into account:

8. Any other comments

Is there anything else you think would be important for a scientist taking on a dog team, or a dog team beginning work on a moss detection project to know:

## Appendix B

**Table A1:** Variables checklist [7].

Key Variable	Relevant Questions	Answers from This Study
Efficacy	Have some measures of sensitivity, precision, and/or specificity been measured as part of this study?	Yes, 100% precision and 100% sensitivity in line-up trials, including the target species as well as other similar moss species.
	Have there been any other measures used to show that the dog is demonstrating the desired effect of their training?	Timed trials took place in the field during which the dogs found the target moss species.
Indication	What form of indication is the dog using (e.g., sit, lie down, point, etc.), and approximately at what distance has the dog been trained to indicate from the target?	Point and stare (stand and stare) for a minimum of 5 s. Indicated immediately when odour first identified. The dog was also in training for curlew nest detection during this project.
	Why was the chosen form of indication considered appropriate for the target species?	We no longer recommend this style of indication; we suggest using a dog that pinpoints rather than indicates from afar. It was suitable during line-ups but proved problematic in the field.
	Consider and provide clear information regarding whose indication performance is being assessed: the dog’s, the team’s or both.	In the discrimination trials, only the dog’s performance was being tested. In the field trials, the team as a whole was being assessed.
Training Samples	What are the training samples/what are the training samples composed of?	The target moss, the discrimination mosses (five different moss species), and distraction odours of grasses, rushes, water samples, Ziploc bags and gloves.
	Where and how were training samples sourced?	They were collected from Slieve Croob, Co. Down, with the help of a bryologist; once located, the handler/trainer collected them using a new pair of nitrile gloves each time.
	How many individual training samples were used across the training stage?	5 samples of the target odour species, 3 samples of each discriminator species.
	How often were training samples reused during training?	This information was not collected.
	How were training samples stored/handled before, during and after training sessions?	The dog handler collected the samples in the presence of a bryologist, using nitrile gloves and jars sterilised by boiling. Samples in jars of the same species were kept together in a Ziploc bag to reduce possible contamination from other samples. These were then stored in an insulated, unheated building that keeps a consistently cool temperature.
Testing Samples	What were the testing samples composed of and were they different to those used in training? If not, why not?	Different samples than those used for training were used; there were three new samples of each of the moss species used for testing.
	Were discrimination trials conducted and if so, why were the selected samples/materials chosen, how similar were they to the target odour, and did any randomization of sample use during trials occur?	The discrimination mosses were all from the same habitat, but some were more closely related than others. Randomisation of pot selection and sample selection was implemented as much as possible.
Blinding	How was blinding implemented in this study and what forms were used?	The discrimination trial was double blind; the tester was out of view of the dog and handler. The handler also continued walking for 5 s after the dog indicated, to avoid influencing the dog’s decision. In the field study, the handler, dog and technician had no knowledge of the location of the moss species, and finds were confirmed by a bryologist who visited the site later in the month.
	During the training and testing stages for the dog, was any form of blinding used, in particular, double blinding during the last stages of training and for testing? If not, why not?	Yes. Blinding was incorporated for some training stages and all testing stages.
Odour Level	Has the dog been trained to find a variety of odour levels? If so, do these odour levels represent what the dog will be looking for in the field?	The dog was trained for the majority of time on a handful of moss, but whilst in the field indicated on larger colonies of moss and alerted on smaller samples showing that the dog was able to generalise quite easily.
Other Target Odours	Have the CDDs used in this study been previously trained on other target odours? If so, which odours and could these species be in the study environment?	Yes, bat and bird carcass for wind turbines, but these would be easily identified and dismissed if located whilst searching for the target moss. The dog was also in training for curlew nest detection at the same time as this project took place.
Dog Selection	Regarding the dog(s) selected for this study, how was this done and why the selected dog(s)?	It was presumed that this would be a harder odour that would requiring a slower, more methodical dog, which this dog was. After the field trial it is now thought that this odour is easier and larger than previously expected, and a dog that pinpointed the target would be more suitable.
	Please provide information about the breeds, personality traits, diets, ages and overall health of the dog(s) selected.	A German Shorthaired Pointer, 5 years old, food and toy driven, good health with sign-off from physio and vet as fit for work. Active working bat and bird carcass detection dog.
Operational Experience	What experience do those training the dog (e.g., dog training, species expert, etc.) and the dog team have?	Main dog trainer/handler—5 years’ experience training and working conservation detection dogs, with 15 years’ experience in conservation. Assistant dog trainer/handler—2 years’ experience training and working conservation detection dogs, with 3 years’ experience training dogs in general, with animal behaviour academic background.
	Has the trainer(s) previously worked in conservation or a field similar to the specific project requirements?	Yes.
	How long has this CDDHT been operational? Has this handler worked with this dog previously? Has the dog had previous successful finds on other species?	Over a year as a team on wind farm projects, having successful live finds of both bats and bird carcasses.
	Is the CDDHT used to working in the specific study environment? Depending on study requirements, have they been trained to do the length of searches asked of them?	Yes, see above experience.
Environment	Please provide a record of the temperature, humidity, wind speed, vegetation density, and precipitation before and during any searches.	Accurate information was not recorded; vegetation density was moderate. The team did not work in extreme weather, including temperature and precipitation.
Search	Overall, how was the search conducted? Please include information regarding the use of a lead, search distance and search time.	Searches were conducted on long line and harness, and the search time was restricted to 40 min, by which time the dog would be rested.
